# Mediators of Allergic Asthma and Rhinosinusitis

**DOI:** 10.1155/2017/7405245

**Published:** 2017-10-04

**Authors:** Young Hyo Kim, Tsuguhisa Nakayama, Da-Tian Bau

**Affiliations:** ^1^Department of Otorhinolaryngology-Head and Neck Surgery, Inha University College of Medicine, Incheon, Republic of Korea; ^2^Department of Otolaryngology-Head and Neck Surgery, Stanford University School of Medicine, Stanford, CA, USA; ^3^Department of Otorhinolaryngology, Jikei University School of Medicine, Tokyo, Japan; ^4^China Medical University, Taichung, Taiwan

The pathophysiologic mechanism of allergic asthma and rhinosinusitis is so complicated and involves thousands of inflammatory mediators. They are world-wide health problems, with its steadily increasing prevalence and its negative impact on quality of life. In spite of many studies throughout the world, there are still much more pathophysiologic mechanisms to be uncovered and the current mainstream of medication still remains symptom control including bronchodilators such as *β*2 agonists, inhaled and systemic corticosteroid, antihistamines, and leukotriene modifiers, all only with transient symptomatic relief. Therefore, we should focus on a novel mechanism and mediators so that we could better understand and cure allergic asthma and rhinosinusitis.

S. Palikhe et al. evaluated a role of the ABCC4 gene polymorphism in airway inflammation of asthmatics. They investigated the potential associations between ABCC4 gene polymorphisms and asthma phenotype. In total, 270 asthma patients and 120 normal healthy controls were enrolled for a genetic association study. As a result, asthmatic patients carrying the G allele at −1508A>G had significantly higher serum levels of periostin, myeloperoxidase, and urinary levels of 15-hydroxyeicosatetraenoic acid and sphingosine-1-phosphate compared with noncarrier asthma patients. Luciferase activity was significantly enhanced in human epithelial A549 cells harboring a construct containing the −1508G allele. Therefore, they concluded that a functional polymorphism in the ABCC4 promoter, −1508A>G, may increase extracellular 15-hydroxyeicosatetraenoic acid, sphingosine-1-phosphate, and periostin levels, contributing to airway inflammation in asthmatics.

D. W. Kim et al. aimed to investigate the role of IL-25 in allergic rhinitis patients sensitized to house dust mite. IL-25 expression in the nasal mucosa from control, nonallergic rhinitis patients, and patients with allergic rhinitis sensitized to house dust mite was assessed using immunohistochemistry (IHC), double IHC, and quantitative reverse transcription PCR. Correlations between IL-25 and other inflammatory markers were also explored.

They observed significantly elevated concentrations of IL-25 in the human nasal epithelial cell samples with the highest doses of mite extracts. Nasal tissues from allergic patients sensitized to mites showed significantly higher IL-25 expression, compared to those from the control or nonallergic patients. They also found that the expression of IL-25 in nasal tissues from allergic patients was positively associated with Th2 immunity markers, such as ECP and GATA3. Therefore, they concluded that IL-25 expression increased with high-dose house dust mite stimulation and it was associated with Th2 immunity markers.

A. Jia et al. evaluated the regulatory role of MBD2 in regulating Th17 cell differentiation and experimental severe asthma by affecting IRF4 expression. As so little is known about that epigenetic regulation of MBD2 in both immunological pathogenesis of experimental severe asthma and CD4^+^ T cell differentiation, they first aimed to establish a neutrophil-predominant severe asthma model, characterized by airway hyperresponsiveness (AHR), BALF neutrophil granulocyte (NEU) increase, higher NEU and IL-17 protein levels, and more Th17 cell differentiation. In the model, MBD2 and IRF4 protein expression increased in the lung and spleen cells. Under overexpression or silencing of the MBD2 and IRF4 genes, the differentiation of Th17 cells and IL-17 secretion showed positive changes. IRF4 protein expression showed a positive change with overexpression or silencing of the MBD2 gene, whereas there was no significant difference in the expression of MBD2 under overexpression or silencing of the IRF4 gene. These data provide novel insights into epigenetic regulation of severe asthma.

H.-M. Lee et al. evaluated the role of a chemical chaperone of endoplasmic reticulum by inhibiting epithelial-mesenchymal transition induced by TGF-*β*1 in airway epithelium via the c-Src pathway. They investigated the role of endoplasmic reticulum (ER) stress and c-Src in TGF-*β*1-induced epithelial-mesenchymal transition (EMT). A549 cells, primary nasal epithelial cells (PNECs), and inferior nasal turbinate organ cultures were exposed to 4-phenylbutylic acid (4PBA) or PP2, and then stimulated with TGF-*β*1. They found that E-cadherin, vimentin, fibronectin, and *α*-SMA expression were increased in nasal polyps compared to inferior turbinates. TGF-*β*1 increased expression of EMT markers such as E-cadherin, fibronectin, vimentin, and *α*-SMA and ER stress markers (XBP-1s and GRP78), an effect that was blocked by PBA or PP2 treatment. 4-PBA and PP2 also blocked the effect of TGF-*β*1 on the migration of A549 cells and suppressed TGF-*β*1-induced expression of EMT markers in PNECs and organ cultures of inferior turbinate.

J. Encarnación-Medina et al. studied the association between selective ATP-binding cassette subfamily C gene expression and proinflammatory mediators released by 2 BEAS-2B after PM_2.5_, budesonide, and cotreated exposures. They assessed ABCC1–4 gene expression changes and proinflammatory cytokine (IL-6, IL-8) release in human bronchial epithelial cells (BEAS-2B). A real-time PCR assay revealed that ABCC1 was upregulated in BEAS-2B exposed after 6 to 7 hr to PM_2.5_ extract or budesonide but downregulated after 6 hr of the cotreated exposure. ABCC3 was downregulated after 6 hr of budesonide and upregulated after 6 hr of the cotreated exposure. ABCC4 was upregulated after 5 hr of PM_2.5_ extract, budesonide, and the cotreated exposure. So they concluded that cotreatment showed an opposite effect on exposed BEAS-2B as compared with budesonide.

H. Park et al. studied the potential biomarkers for NSAID-exacerbated respiratory disease. NSAID-exacerbated respiratory disease (NERD) is an endotype characterized by asthma, chronic rhinosinusitis (CRS) with nasal polyps, and hypersensitivity to aspirin/cyclooxygenase-1 inhibitors. NERD is more associated with severe asthma than other asthma phenotypes. In this review, they summarized the known potential biomarkers of NERD that are distinct from those of aspirin-tolerant asthma and they also provided an overview of the different NERD subgroups.

Finally, Y. Choi et al. evaluated the role of neutrophil extracellular DNA traps in inducing autoantigen production by airway epithelial cells. They aimed to prove that neutrophil extracellular DNA traps (NETs), cytotoxic molecules released from neutrophils, are a key player in the stimulation of airway epithelial cells (AECs) to produce autoantigens. This study showed that NETs significantly increased the intracellular expression of tissue transglutaminase (tTG), but did not affect that of CK18 in AECs. NETs induced the extracellular release of both tTG and CK18 in a concentration-dependent manner. Moreover, NETs directly degraded intracellular *α*-enolase into small fragments. However, antibodies against neutrophil elastase (NE) or myeloperoxidase (MPO) attenuated the effects of NETs on AECs. Furthermore, each NET isolated from healthy controls (HC), nonsevere asthma (NSA), and SA had different characteristics.

I hope that by reading this special issue, you will get not only the latest insights into mediators related to the pathophysiology of allergic asthma and rhinosinusitis but also an opportunity to get a good idea of motivating your recent research.

